# Showering Emboli of an Atrial Mass: A Fatal Phenomenon

**DOI:** 10.5812/cardiovascmed.9392

**Published:** 2013-05-20

**Authors:** Anita Sadeghpour, Azin Alizadehasl

**Affiliations:** 1 Echocardiography Research Center, Rajaie Cardiovascular Medical and Research Center, Tehran University Medical Center, Tehran , IR Iran; 2 Department of Cardiovascular, Cardiovascular Research Center, Tabriz University of Medical Sciences, Tabriz, IR Iran

**Keywords:** Atrial myxoma, familial, Embolism

*Dear Editor*

Left atrial (LA) myxoma, the most common type of primary cardiac tumor is an uncommon disease with an annual incidence of 0.5 per million populations which may be associated with brief isolated ischemic events or may represent a major source of neurological morbidity. Clinically apparent cerebral emboli have been reported in the range of 25%. In most patients with atrial myxoma, embolization of tumor fragments results clinically in isolated nonfatal ischemic strokes, but in others sudden plugging of the mitral valve opening may cause syncope or sudden cardiac death. So although atrial myxomas are rare, failure to recognize them may deprive patients of life-saving tumor resection (1-3).

We describe a 73-year-old woman with history of confirmed diagnosis of LA mass (max size = 3x4cm) highly suggestive of LA myxoma by transesophageal echocardiography (TEE) two months ago. It had a gelatinous appearance with villous surface. She refused surgery at that time and presented with a rapidly progressive illness resulting in coma within 24 hours from multiple myxomatous emboli. On arrival to the emergency room, physical examination revealed shallow breathing with a respiratory rate of 29/min, blood pressure of 180/110 mm Hg, and temperature of 37.7°C. She was unresponsive to verbal stimuli and had no spontaneous motor movements. Eyes were deviated to the right. With painful stimuli she grimaced. Pupils were 3 mm in size and minimally reactive to light. No papill-edema was found. Muscle-stretch reflexes were increased in both the upper and lower extremities with bilateral extensor responses.

Laboratory examination revealed a respiratory, compensated metabolic acidosis with a lactate level of 7 mmol/L. Creatinine phosphokinase and Troponin I were very high. Levels of urea nitrogen and creatinine were increased most probably due to tumor emboli in kidney artery. The electrocardiogram showed a normal sinus rhythm with acute ST segment elevation inferolateral myocardial infarction due to embolization in coronary artery (3). A radiograph of the chest showed near normal findings. Cranial computed tomographic scan revealed several low attenuation areas within both cerebral hemispheres with multiple hemorrhages scattered throughout both hemispheres and effacement of cerebral-sulci, consistent with massive cerebral infarction. The patient rapidly progressed to unresponsive coma. Also cold and pulseless left arm and both lower extremities were highly suggestive of multiple peripheral artery embolizations (2, 3). Surprisingly TEE showed no any remenant of LA mass, large patent foramen ovale (PFO) and large emboli in proximal portion of right pulmonary artery (simultaneous pulmonary emboli) with moderate both ventricular dysfunction.

Previous studies suggest that atrial myxoma is seen in 0.5% of acute stroke patients, with women in the fifth decade at greatest risk. Transthoracic echocardiograms have been repeatedly negative in patients with atrial myxoma, TEE or magnetic resonance imaging may increase the detection of atrial myxoma (4-6). Atrial myxoma may present with many clinical syndromes. Patients are seen primarily by internists or cardiologists with palpitations, exertional dyspnea, fever, fatigue, or weight loss or syncope. In addition, the exceptional friable and spongy mass of atrial myxoma may easily dislodge fragments into the systemic circulation. The central nervous system is one of the most susceptible areas of embolization (1, 5, 6).

Our patient arrived at the emergent departement in a moribund condition and demonstrated an unrelenting process of embolization. An overwhelming shower of myxomatous material resulted in occlusion of many arteries even pulmonary artery via PFO. More frequently, patients with atrial myxoma are seen with small and single territory of cerebral infarcts, this much vessels occlusion has not yet been reported, to our knowledge. Our case was unique regarding to its gelatinous appearance and villous surface LA myxoma resulting in fatal multiple organ embolizations. It can be concluded that large gelatinous myxoma with villous surface should be surgically resected as soon as possible specially if associated with PFO.
